# Hybrid care in mental health: a framework for understanding care, research, and future opportunities

**DOI:** 10.1038/s44277-024-00016-7

**Published:** 2024-10-17

**Authors:** Kelly Chen, Jack J. Huang, John Torous

**Affiliations:** 1 Beth Israel Deaconess Medical Center, Harvard Medical School, Boston, MA USA; 2 Brigham and Women’s Hospital, Harvard Medical School, Boston, MA USA

**Keywords:** Health care, Health services, Outcomes research

## Abstract

Technology is playing an increasing role in healthcare, especially in mental health. Traditional mental healthcare, whether in-person or via telehealth, cannot by itself address the massive need for services. Standalone technology such as smartphone apps, while easily accessible, have seen limited engagement and efficacy on their own. Hybrid care – the combination of synchronous in-person or telehealth appointments with the use of asynchronous digital tools such as smartphone applications, wearable devices, or digital therapeutics – has the potential to offer the best of both worlds, providing both increased access and higher engagement and efficacy. In this paper, we present a framework highlighting the key components of hybrid care models: digital intervention, human support, and target population. This framework can be used to evaluate existing models in the literature and in practice, identify areas of need and opportunity, and serve as a blueprint for key elements to consider when designing new hybrid care models.

## Lay summary

This perspective dissects three main dimensions of hybrid care: intervention, human support, and clinical target for evaluating current research and future opportunities in the space.

## Introduction

The use of technology in healthcare is rapidly increasing, especially in mental health. COVID-19 accelerated the uptake of telemedicine for mental health, and national 2023 data highlights that virtual visit rates remain much higher in mental health than in other specialties [1]. While synchronous care provided through real-time video visits or phone calls can increase access to clinicians, they do not necessarily increase access to care overall, as there remains a limited number of clinicians. Thus other forms of telehealth, in the form of asynchronous telehealth technology such as self-guided smartphone apps, virtual reality (VR) tools, and more recently large language model (LLM) chatbots, are of great interest [2, 3]. Asynchronous modes of intervention are technologies that can be leveraged and used independently or in support of care both outside and/or during clinical sessions. With smartphone ownership at nearly 90% [4], the potential for anyone to download an app and access evidence-based care tools is an appealing vision. However the realization that well over 90% of people stop using a mental health app within a few days [5] has created doubt about the future of this approach.

Addressing the access issues of traditional telehealth while maintaining its efficacy and addressing engagement issues of asynchronous telehealth while maintaining its accessibility need not be a binary decision. There is an opportunity for a third approach – hybrid care – which combines the therapeutic relationship and personalized touch of traditional mental healthcare (whether in-person or telehealth) with the scalability and efficiency of asynchronous digital technology. In this article, we seek to define hybrid care in mental health as well as share a framework for approaching hybrid care solutions. This framework highlights key components of hybrid care programs that are critical to examine when evaluating a hybrid care model in the literature or in practice. This framework can also help identify areas of need and opportunity and provide a starting point for designing new hybrid care solutions.

**Fig. 1:**
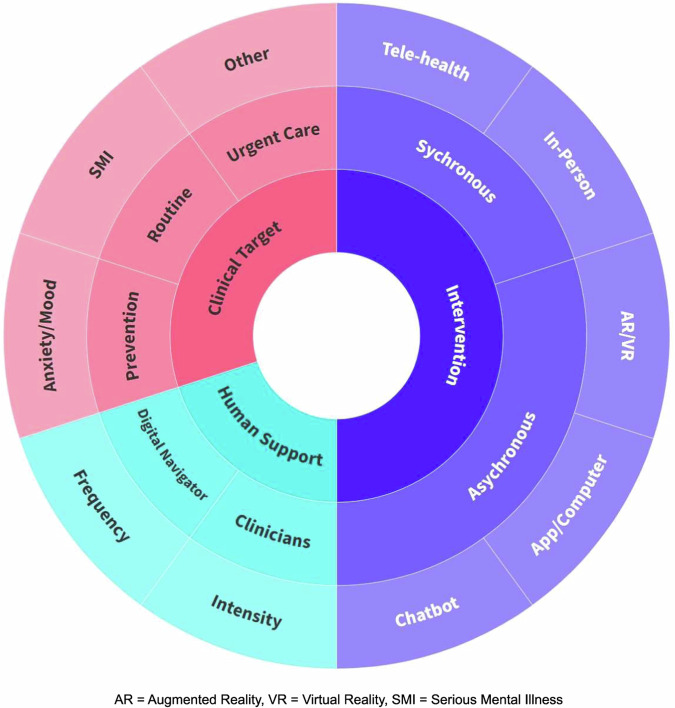
The three main dimensions of hybrid care caption. The different colors of this figure help to denote the three main dimensions of hybrid care discussed in this work. Starting in the middle of the figure, this figure serves as a guide to the different combinations of clinical targets, human supports, and interventions leveraged across different hybrid care models. The three main dimensions of hybrid care discussed here are in the innermost circle: intervention, human support, and clinical target. Moving outwards, the middle ring represents different divisions of the three main dimensions of hybrid care. For interventions, this can be provided synchronously in-person, or through telehealth as listed in the outermost ring. There are also asynchronous hybrid care interventions, and these include smartphone apps, computers, chatbots, and artificial intelligence (AR)/virtual reality (VR). The human support dimension of hybrid care includes the digital navigator and the clinicians both of which can vary in frequency and intensity of support. The clinical target dimension of hybrid care represents the different goals, or the type of care being provided and which population it’s being provided to, whether that be routine therapy for those with SMI, anxiety, or depression, or urgent care for other disorders.

First, it is important to define hybrid care. As the name suggests, hybrid care models combine traditional face-to-face or telehealth appointments with the use of digital tools, such as smartphone applications, wearable devices, or digital therapeutics, to enhance and personalize care delivery. Sometimes also called blended care, hybrid care models extend traditional visits (be they in-person or online) with the use of asynchronous telehealth between these visits. While simple in concept, the design and quality of these hybrid care models can vary widely. Understanding three key dimensions (Fig. 1) that influence the design, implementation, and evaluation of hybrid care models is key to understanding the advantages of different models and the applicability of that model for use in local settings.

## Dimension 1: intervention

While all hybrid care models offer a combination of live synchronous clinical sessions and asynchronous digital interventions that may or may not be live or in the office, these can both vary in mode of delivery. Therapy sessions can take place both in-person and online in a hybrid model; it is important to note that telehealth sessions have maintained a higher rate of popularity compared to other specialties after the pandemic [1]. And while different variations of cognitive behavioral therapy (CBT), such as for cognitive behavioral therapy for psychosis (CBTp) [6], have been very popular in the current literature, other types of psychotherapy can also be utilized in hybrid care, such as dialectical behavior therapy (DBT) [7]. The asynchronous digital intervention as depicted in the right half of Fig. 1 can vary widely as well. The most common asynchronous tool is a symptom-monitoring or self-help CBT smartphone app used between sessions [8]. These applications can help patients gain greater insight into their symptoms and triggers and practice skills outside of synchronous sessions, while also collecting data that clinicians can use to better inform treatment. With the emergence of new technologies and artificial intelligence (AI), there are also examples of virtual reality (VR), augmented reality (AR), and chatbots as part of hybrid care models [9, 10]. For example, VR/AR tools can provide realistic simulations to aid in therapy for trauma or anxiety disorders, and chatbots can provide support in between clinical sessions. With thousands [8] of mental health apps and monthly changes in what chatbots can offer, there are a number of evolving choices of asynchronous interventions. Resources to help guide this selection include the American Psychiatric Assocation (APA) App evaluation framework [11] and the mindapps.org database which contains hundreds of smartphone apps for mental health and wellness that are incompliance with the APA’s app evaluation framework [12]. While there is still room for improvement for the apps available, this database can help separate the more optimal tools.

Presently, computer and smartphone apps are the most commonly used digital tools in the hybrid care space, due to both the ubiquity of smartphones and the ability of apps to both collect data and deliver interventions such as CBT. Several factors differentiate smartphone apps that may be utilized in hybrid care. Some hybrid care models use a third-party, commercially available app, whereas other models develop their own applications in-house. Apps developed with and for the patient population of interest are generally more well-received [13], although creating a new application can also pose challenges. Smartphone apps may collect data from users either actively (user directly enters information such as their symptoms) or passively (the app collects sleep data, etc). This can serve as the basis for digital phenotyping and delivering just-in-time interventions, either through the app or other channels.

## Dimension 2: human support

Hybrid care is designed to harness the efficiency of technology, but the majority of hybrid care models still utilize some degree of human support (bottom left section of Fig. 1). Firstly, the clinician provides therapies to the patient and the intensity such as type of therapy provided can vary as provided by the flexibility of hybrid care. Secondly, additional human support can be targeted toward patients, providers, or both. From the patient perspective, some amount of human support is often critical to driving engagement with technology. While certain aspects of digital interventions may by themselves encourage engagement (e.g., apps that review symptoms recorded or provide platforms for therapy homework), having live human support such as a coach who checks in and reviews progress can further encourage patients to engage. Human support can also help patients navigate digital platforms and thereby can be key to promoting equity for populations with lower digital literacy. A similar role can be helpful for providers too; having a support person who reviews data and summarizes changes may increase the utility of and engagement with technology. For either patients or providers, this additional human support may be done via video or phone calls, text messages, emails, in-person sessions, or a combination thereof.

Hybrid care models can provide human support either through a dedicated digital support role or through the clinicians themselves. The Digital Navigator model offers one example of a dedicated technology support role that can be customized for local use cases. Described in more detail elsewhere [14], the Digital Navigator is a non-clinician trained to bridge the digital literacy gap for clinicians and patients by removing the burdens of digital tool integration for clinicians and providing direct technology support to patients as needed. The frequency of support provided by the digital navigator can also be tailored to the needs of the patients; patients who need more help with digital tools can receive a higher frequency of appointments compared to those who are more digitally literate or need less support. While some hybrid care models place the responsibility of digital support onto the clinicians themselves [15–17], many models have found a dedicated digital support role to be helpful [18, 19]. Currently, there is limited evidence as to the optimal level of training, mode of contact, and frequency of interactions for the digital support role, but each of these variables is important to consider when evaluating and implementing hybrid care models.

## Dimension 3: clinical target

The third key dimension to consider in evaluating hybrid care models is the target population. Hybrid care models may target different phases of care, from prevention to routine to urgent care, and address a variety of clinical conditions, including mood and anxiety disorders, serious mental illness (SMI), and others (upper left section of Fig. 1). Hybrid care models have often focused on mood and anxiety disorders, with most patients experiencing mild to moderate levels of depression and anxiety [2]. Patient acuity and severity in hybrid care studies tend to skew toward the lower end; many models and studies in the literature exclude patients who demonstrate acute suicidality [20, 21]. Nonetheless, literature is now emerging on hybrid care models targeting patients with serious mental illness such as psychotic-spectrum disorders [22]. Such studies are critical for expanding the potential impact of technology to patients with serious and severe mental illness, who often face the greatest challenges in accessing care.

Along with determining the target population of a hybrid care model, it is also important to consider the actual patient population that is selected for and engages with hybrid care. Patient recruitment methods for hybrid care models vary, with some studies utilizing physician referrals [17, 23] and others relying on self-referrals by social media ads [15, 18]. Especially in the case of self-referral, patients may have clinical conditions, severity, and willingness to engage with technology that differ from the target population, and as such, any outcomes from such studies may not be generalizable. Even when patients are referred by physicians, the patients who engage with hybrid care may still represent a biased subset of the target population. The severity of a patient’s illness may also influence their choice of intervention, potentially skewing results. For example, one study showed that patients undergoing more intensive interventions (featuring technology, medication, and therapy) had lower rates of remission than those undergoing less intensive interventions, but this was likely been driven by individuals with greater severity of symptoms self-selecting for more intensive interventions, rather than more intensive interventions being less effective [2]. For all these reasons, it is critical to understand both the target population and the actual population that engages with a hybrid care program, especially when evaluating outcomes.

## Discussion

The three key factors discussed above - digital intervention, human support, and clinical target – underpin the design and implementation of hybrid care models. We hope that this framework, illustrated in Fig. 1, serves several key purposes. First, this framework serves as a guide when evaluating existing hybrid care programs, whether in literature or in practice. The components we highlight are defining features of hybrid care programs that influence their use cases, efficacy, and application in different settings. Second, this framework can help identify areas of need and opportunity. For example, many existing hybrid care models use a smartphone app or computer interface for routine care of mild to moderate anxiety or depression [19, 24]. Other areas as illustrated in the figure, such as serious mental illness, preventative psychiatry, or the use of innovative technologies, present areas of future opportunity. Third, our framework provides a roadmap for key factors to consider when developing a hybrid care program. While not necessarily exhaustive, the components in Fig. 1 are key decision points to consider when designing a hybrid care model.

### Future directions and opportunities

Existing digital navigator trainings can be more widely adopted, and the digital navigator role can be integrated as a separate support role in care. As healthcare increasingly incorporates digital tools, these digital navigators can help bridge the gap for patients who may be facing challenges with digital literacy or even technology access. This role can provide personalized support in helping patients leverage digital tools to support their care. While the process of reimbursement from insurance companies is still evolving, with its current challenges in unclear billing codes for digital tools, growth in this area could help more patients access digital tools as a part of routine care.

## Conclusion

The mental healthcare sector is in the midst of a shift towards the integration of technology, starting with the surge of telehealth adoption and extending to the increasing use of digital interventions. Hybrid care has the potential to capitalize on the strengths of both traditional and digital approaches to mental healthcare while addressing the limitations of each. When designing hybrid care models or evaluating studies in the literature, it is critical to examine the key domains of the digital intervention being utilized, the nature of human support, and the patient population being targeted by and that engages with the program. As technology rapidly advances, the reach of hybrid care will only increase. These key components of hybrid care models provide a framework for designing and evaluating programs to ensure the efficient, equitable, and efficacious application of technology in shaping mental healthcare delivery.

### Citation diversity statement

The authors have attested that they made efforts to be mindful of diversity in selecting the citations used in this article.

## References

[1] Bartelt K, Piff A, Allen S, Barkley E. Telehealth utilization higher than pre-pandemic levels, but down from pandemic highs. Epic Res. https://epicresearch.org/articles/telehealth-utilization-higher-than-pre-pandemic-levels-but-down-from-pandemic-highs. Accessed 18 June 2024.

[2] Nelson BW, Peiper NC, Forman-Hoffman VL. Digital mental health interventions as stand-alone vs. augmented treatment as usual. BMC Public Health. 2024;24:969. 38580986 10.1186/s12889-024-18412-1 PMC10998421

[3] Rus-Calafell M, Schneider S. Are we there yet?!—a literature review of recent digital technology advances for the treatment of early psychosis. Mhealth. 2020;6:3. 32190614 10.21037/mhealth.2019.09.14 PMC7063271

[4] Sidoti O, Gelles-Watnick R, Faverio M, Atske S, Radde K, Park E. Mobile Fact Sheet. Pew Research Center. 2024. https://www.pewresearch.org/internet/fact-sheet/mobile/. Accessed 18 June 2024.

[5] Baumel A, Muench F, Edan S, Kane JM. Objective user engagement with mental health apps: systematic search and panel-based usage analysis. J Med Internet Res. 2019;21:e14567. 31573916 10.2196/14567 PMC6785720

[6] Garety P, Ward T, Emsley R, Greenwood K, Freeman D, Fowler D, et al. Effects of SlowMo, a blended digital therapy targeting reasoning, on paranoia among people with psychosis: a randomized clinical trial. JAMA Psychiatry. 2021;78:714–25. 33825827 10.1001/jamapsychiatry.2021.0326 PMC8027943

[7] Rizvi SL, Dimeff LA, Skutch J, Carroll D, Linehan MM. A pilot study of the DBT coach: an interactive mobile phone application for individuals with borderline personality disorder and substance use disorder. Behav Ther. 2011;42:589–600. 10.1016/j.beth.2011.01.003 22035988

[8] King DR, Emerson MR, Tartaglia J, Nanda G, Tatro NA. Methods for navigating the mobile mental health app landscape for clinical use. Curr Treat Options Psychiatry. 2023;10:72–86. 10.1007/s40501-023-00288-4 PMC10206563 37360961

[9] Carlson CG. Virtual and augmented simulations in mental health. Curr Psychiatry Rep. 2023;25:365–71. 37624512 10.1007/s11920-023-01438-4

[10] Haque MR, Rubya S. An overview of chatbot-based mobile mental health apps: insights from app description and user reviews. JMIR mHealth uHealth. 2023;11:e44838. 37213181 10.2196/44838 PMC10242473

[11] Henson P, David G, Albright K, Torous J. Deriving a practical framework for the evaluation of health apps. Lancet Digit Health. 2019;1:e52–4. 33323229 10.1016/S2589-7500(19)30013-5

[12] Camacho E, Cohen A, Torous J. Assessment of mental health services available through smartphone apps. JAMA Netw Open. 2022;5:e2248784. 36576737 10.1001/jamanetworkopen.2022.48784 PMC9857226

[13] van Kessel R, Roman-Urrestarazu A, Anderson M, Kyriopoulos I, Field S, Monti G, et al. Mapping factors that affect the uptake of digital therapeutics within health systems: scoping review. J Med Internet Res. 2023;25:e48000. 37490322 10.2196/48000 PMC10410406

[14] Chen K, Lane E, Burns J, Macrynikola N, Chang S, Torous J. The digital navigator: standardizing human technology support in app-integrated clinical care. Telemed e-Health. 2024;30:e1963–70. 10.1089/tmj.2024.0023 38574251

[15] Economides M, Ranta K, Nazander A, Hilgert O, Goldin PR, Raevuori A, et al. Long-term outcomes of a therapist-supported, smartphone-based intervention for elevated symptoms of depression and anxiety: quasiexperimental, pre-postintervention study. JMIR mHealth uHealth. 2019;7:e14284. 31452521 10.2196/14284 PMC6733157

[16] Buelens F, Luyten P, Claeys H, Van Assche E, Van Daele T. Usage of unguided, guided, and blended care for depression offered in routine clinical care: lessons learned. Internet Interv. 2023;34:100670. 37767005 10.1016/j.invent.2023.100670 PMC10520335

[17] Macrynikola N, Nguyen N, Lane E, Yen S, Torous J. The digital clinic: an innovative mental health care delivery model utilizing hybrid synchronous and asynchronous treatment. NEJM Catal Innov Care Deliv. 2023;4:CAT-23. 10.1056/CAT.23.0100.

[18] Camacho E, Chang SM, Currey D, Torous J. The impact of guided versus supportive coaching on mental health app engagement and clinical outcomes. Health Inform J. 2023;29:14604582231215872. 10.1177/14604582231215872 38112116

[19] Wolitzky-Taylor K, LeBeau R, Arnaudova I, Barnes-Horowitz N, Gong-Guy E, Fears S, et al. A novel and integrated digitally supported system of care for depression and anxiety: findings from an open trial. JMIR Ment Health. 2023;10:e46200. 37486735 10.2196/46200 PMC10407647

[20] Owusu JT, Wang P, Wickham RE, Varra AA, Chen C, Lungu A. Real-world evaluation of a large-scale blended care-cognitive behavioral therapy program for symptoms of anxiety and depression. Telemed e-Health. 2022;28:1412–20. 10.1089/tmj.2021.0590 PMC9587796 35263185

[21] Graham AK, Greene CJ, Kwasny MJ, Kaiser SM, Lieponis P, Powell T, et al. Coached mobile app platform for the treatment of depression and anxiety among primary care patients: a randomized clinical trial. JAMA psychiatry. 2020;77:906–14. 32432695 10.1001/jamapsychiatry.2020.1011 PMC7240649

[22] Cohen M, Roe D, Savir T, Baumel A. Blended care in psychosis–A systematic review. Schizophr Res. 2024;267:381–91. 38636358 10.1016/j.schres.2024.03.041

[23] Possemato K, Kuhn E, Johnson E, Hoffman JE, Owen JE, Kanuri N, et al. Using PTSD Coach in primary care with and without clinician support: a pilot randomized controlled trial. Gen Hosp Psychiatry. 2016;38:94–8. 26589765 10.1016/j.genhosppsych.2015.09.005

[24] Roos LG, Sagui-Henson SJ, Castro Sweet C, Welcome Chamberlain CE, Smith BJ. Improvement and maintenance of clinical outcomes in a digital mental health platform: findings from a longitudinal observational real-world study. JMIR mHealth uHealth. 2024;12:e48298. 38913405 10.2196/48298 PMC11231619

